# Therapist characteristics and acceptance of internet-delivered cognitive behavioral therapy: A national cross-sectional survey using the technology acceptance model after ten years of iCBT in Norway^☆^

**DOI:** 10.1016/j.invent.2025.100881

**Published:** 2025-10-03

**Authors:** Reidar Nævdal, Christiaan Vis, Robin Maria Francisca Kenter

**Affiliations:** a Research Centre for Digital Mental Health Services, Division of Psychiatry, Haukeland University Hospital, Post Office Box 1400, Bergen, N-5021, Norway; b University of Bergen, Department of Global Public Health and Primary Care, Post Office Box 7804, Bergen, N-5020, Norway; c Amsterdam University Medical Centre, Department of Public and Occupational Health and Amsterdam Public Health research institute, Amsterdam, Netherlands

**Keywords:** iCBT, iCBT-therapist, Digital mental health, Therapist acceptance, Technology acceptance model, Implementation

## Abstract

**Background:**

Internet-delivered cognitive behavioral therapy (iCBT) has been available in Norwegian specialist mental health care for a decade. Despite strong evidence and national support, uptake remains limited, with little known about therapists delivering iCBT or factors influencing engagement.

**Objective:**

Using an extended Technology Acceptance Model (TAM), this study aimed to provide a national overview of therapists delivering iCBT, their work environment, and acceptance, as well as explore group differences between clinics established through different implementation approaches.

**Methods:**

The study identified all therapists using iCBT in routine care. Using a cross-sectional survey, data regarding therapists' characteristics, their context, and TAM constructs was collected. Therapists were grouped by whether their clinics used bottom-up or top-down implementation approaches and compared on all TAM constructs.

**Results:**

Sixty-seven therapists were identified as actively delivering iCBT nationally. Of these, 45 therapists from 18 clinics responded (67 % response rate). Therapists were experienced clinicians with varying backgrounds and work environments. High acceptance was found across all TAM dimensions except for Image. Therapists in clinics with structured top-down implementation and ongoing supervision reported highest acceptance.

**Conclusion:**

Despite high therapist acceptance of iCBT, experienced loss in professional regard presents a cultural barrier hindering widespread adoption. Despite a decade of use, iCBT remains a small part of routine care. However, successful implementation across diverse therapist backgrounds is achievable, and mandated top-down implementation appears useful when supported by experienced supervision.

**Trial registration:**

Haukeland University Hospital e-procotol, project ID: 4696–4696.

## Highlights

• First national overview of iCBT therapists in Norwegian specialist health care
• Only 67 therapists deliver iCBT after 10 years of use in routine care
• Therapist acceptance of iCBT was high
• iCBT was not seen as enhancing professional status, suggesting a cultural barrier
• Clinics established through top-down processes with high support had the highest acceptance

## 1 Introduction

Over the last decade, therapist guided Internet-delivered Cognitive Behavioral Therapy (iCBT) has increasingly been implemented within specialist mental healthcare services in several countries, including Norway (Folker et al., 2018; Titov et al., 2018). The main motivation for offering iCBT in routine healthcare is to improve access to evidence-based treatments and increase cost-effectiveness. Although unguided iCBT needs less resources, several countries have opted to implement guided iCBT where a trained therapist supports patients through asynchronous messaging systems or telephone calls (Titov et al., 2018). Indeed, growing evidence indicates that iCBT, particularly when guided by a therapist, can achieve comparable outcomes to face-to-face therapy while requiring fewer clinical resources (Carlbring et al., 2018; Etzelmueller et al., 2020; Hedman-Lagerlöf et al., 2023; Karyotaki et al., 2018; Käll et al., 2024). Digitalization of mental health services, like iCBT, is therefore now a central component of the World Health Organization's strategies for global health (World Health Organization, 2019).

Despite the evidence-base and effort to implement guided iCBT, the uptake in routine mental healthcare remains limited, with success varying across settings (Molloy et al., 2021; Repål et al., 2023; Titov et al., 2018; Vis et al., 2023). These differences are also reflected in how iCBT has been implemented into routine practice. For example, some clinics emerged through *bottom-up* initiatives from staff at a local research group or clinic, while others were established through *top-down* processes, were a higher-level authority initiated or mandated the use of iCBT.

Interestingly, although the therapeutic content and principles in iCBT mirrors traditional cognitive behavioral therapy (Andersson et al., 2016), the difficulty in implementation persists. However, the implementation of iCBT does not just mean implementing the therapeutic principles of CBT, but also the technology used to deliver these principles. In contrast to the therapeutic content, this technology does represent a divergence from traditional face-to-face practice. Successful implementation of guided iCBT therefore requires clinicians to adopt not just a different treatment approach, but also a digital product (Chong et al., 2022; Cresswell and Sheikh, 2013; Garavand et al., 2016). This digital product may give the therapists some advantages compared to face-to-face therapy, but it also limits the therapists approach to the functionality offered by the technology. For example, through an iCBT the therapist may be able to treat a patient that cannot attend face-to-face therapy sessions during the therapists' working hours. On the other hand, if the iCBT contains superfluous information that, in the therapist's mind, is unnecessary for a specific patient, the therapist may still be forced to work through this information with their patients as part of the iCBT. Furthermore, to use an iCBT therapists must also develop novel therapeutic skills, like using asynchronous written messages as their main communication with the patient. While these challenges are likely to affect the adoption of an iCBT, they are not a consequence of the therapeutic approach itself, but the digital product that is used to deliver the treatment.

The implementation of guided iCBT entails that both patient and therapist must interact with a technology system. While several studies exploring the implementation of iCBT focus on patients, less attention have been given to therapists and their role in implementation. Although the patient perspective is important, the therapists are a trusted authority when shared decision-making is done with the patient regarding treatment approach. But more than this, therapists are the ones tasked with delivering the iCBT and their opinions regarding this method ultimately determines whether it is offered at all.

Although the idea of the technology as a distinct element in the implementation of iCBT may be novel in some sense, this idea is studied extensively in the field of technology adoption. Summarizing much of this research, the Technology Acceptance Model (TAM) describes factors influencing the adoption and use of a technology system (Davis, 1989; Davis et al., 1989; Venkatesh and Bala, 2008; Venkatesh and Davis, 2000). According to the model, a person's behavioral intention (BI) to use a technology is a primary determinant of actual usage. BI is influenced by two variables: perceived usefulness (PU), defined as the degree to which a user believes that using a technology will improve their job performance, and perceived ease of use (PEOU), which refers to the degree to which a user perceives the technology as easy to use (Davis, 1989). Later extensions of TAM, including TAM2 (Venkatesh and Davis, 2000) and TAM3 (Venkatesh and Bala, 2008), further refined the model by identifying latent variables that influence PU, PEOU. These latent variables, commonly referred to as “antecedent factors”, were categorized into four groups: individual factors, social influence, facilitating conditions and system characteristics (Venkatesh and Bala, 2008). Individual factors refer to characteristics regarding the user of a technology. In other words, people may differ in their understanding of how useful or easy to use a technology is. In contrast, social influence and facilitating conditions refer to how the user's context may affect their perceptions of the technology through social psychological processes or through support structures which offer resources for the users. Finally, the system characteristics simply refers to the technology itself and implies that some systems may be perceived as easier to use or more useful to a user.

Although the TAM has shown its value in understanding and improving technology adoption across sectors such as education, business, and information systems (Davis et al., 2024; King and He, 2006; Venkatesh, 2000; Venkatesh and Bala, 2008; Venkatesh and Davis, 2000), its application in healthcare, particularly in understanding mental health professionals' engagement with digital tools, has been more limited. At the same time, a fundamental gap in the current knowledge base concerns the lack of any systematic information about the therapists delivering iCBT in routine care—how many they are, what professional backgrounds and characteristics they represent, the organizational contexts in which they work, and the structural conditions that shape their delivery of care is all mostly unknown. Consequently, knowledge about how these individual and contextual factors relate to therapists' acceptance of iCBT remains incomplete. By applying the TAM as a conceptual framework, this study seeks to examine how these constructs manifest among therapists currently delivering iCBT in specialized mental healthcare. Generating this knowledge is essential for informing targeted implementation strategies, optimizing support structures, and enabling the sustainable integration of iCBT into routine mental health care.

### 1.1 Aim

The study aims to provide the first comprehensive description of therapists using iCBT in routine care. Investigating therapists delivering guided iCBT within the Norwegian Specialist Health Services, the study includes therapists' demographic and professional characteristics, and their acceptance of iCBT as measured using an extended TAM. Additionally, the study examines whether acceptance levels differ between iCBT clinics established through different implementation approaches - specifically, early adopting clinics established through bottom-up processes initiated locally, versus clinics established through top-down processes where a centralized and higher-level authority initiated and mandated the use of iCBT in local clinics. Where differences in acceptance are identified, the study explores potential explanatory factors by examining differences in therapist characteristics and organizational factors (such as work arrangements and clinic structure) to better understand what shapes therapist acceptance.

The study has the following research questions: 1) What are the demographic and professional characteristics of therapists delivering iCBT in routine mental health care in Norway? Specifically, how many therapists are currently engaged in iCBT delivery, in which organizational settings do they work, and how can their demographic and professional profiles be characterized? 2) What are these therapists' acceptance levels of iCBT as measured by TAM? 3) Do acceptance levels differ significantly between clinic groups established through different implementation approaches? And 4) if acceptance differences are found, what therapist characteristics and organizational factors distinguish these groups?

## 2 Method

### 2.1 Study design

The study employed a naturalistic, multi-site, cross-sectional survey targeting all iCBT therapists working in specialist mental health services in Norway. The data presented are baseline measures from a larger longitudinal implementation study.

### 2.2 Setting

The study was conducted in the Norwegian specialist health care services, which delivers therapies for patients with psychiatric illnesses through psychiatric hospitals. Originally iCBT in Norway was delivered through one outpatient clinic at a single psychiatric hospital. This began as a research project starting in 2013, before becoming part of routine care in 2015. As opposed to a centralized delivery model where a single iCBT clinic is used to deliver the treatment, Norway has the last decade spread the use of iCBT to several existing outpatient clinics, resulting in a decentralized delivery model. At the time of data collection (February 2024), no official overview of how many clinics there were, what characteristics they had or how many therapists offered iCBT existed.

While an overview of the clinics was lacking, all clinics delivering iCBT in the Norwegian specialist health care services use the same three diagnosis specific guided iCBTs for depression, social anxiety disorder and panic disorder. This entails that the clinics use the same digital platform, with the same treatment content, asynchronous messaging system, symptom measures and treatment structure. As with other treatments in the Norwegian specialist health care services, the standard way of accessing the iCBT treatments is through a referral from a general practitioner (GP). However, some of the iCBT clinics also allowed for self-referral, where the patient could refer themselves to the iCBT clinic independent of a GP (Bjarke et al., 2025). Since they operate within the specialist health care service, all clinics were also obliged to follow the national clinical guidelines of specialist health care in Norway (Helsedirektoratet., 2015).

### 2.3 Participant recruitment and data collection

To recruit the participants, we identified and contacted all team‑leaders in charge of iCBT-clinics in a psychiatric hospital. This was done by reaching out to known team‑leaders and key personnel in charge of supervising either therapists or other team‑leaders. The team‑leaders were informed about the study and its goals, as well as what participating entailed. They were asked to inform the iCBT-therapists employed at their clinic about the study and supply their email-addresses. All leaders responded, giving the study a complete list of all iCBT therapists at the time (February 2024),

Information about the study and a link to informed consent forms as well as a survey was sent to all supplied email addresses in February 2024. Acknowledging that clinicians working in the specialist health services are pressed for time, the survey was kept open for therapists to answer for five weeks till the end of March 2024. During this time a weekly reminder email was sent to those who had not found the time to reply.

### 2.4 Measures

#### 2.4.1 Conceptual framework

**Fig. 1:**
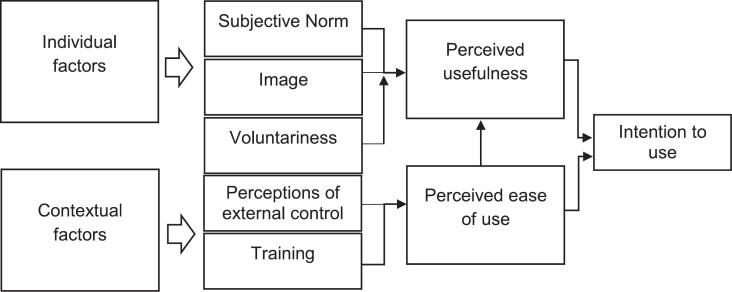
The extended technology acceptance model used in the study.

To explore how therapists perception of iCBT as a technology system may affect the implementation of iCBT, the study applied an extension of the TAM (Davis, 1989) as a conceptual framework (see Fig. 1). Rather than testing the full set of hypothesized relationships between model components as done in previous TAM research (Davis et al., 2024), this study used the TAM to structure and select which variables to measure and analyze. The TAM has been widely applied to understand technology adoption across various contexts, and its core constructs—perceived usefulness, perceived ease of use, and behavioral intention to use—are considered robust predictors of individual acceptance of digital tools.

The extended TAM applied in the study included contextual and individual-level variables relevant to therapists working in specialist mental health services with iCBT. This approach enabled a deeper understanding of how therapists engage with iCBT in real-world clinical settings, and what factors may support or impede its broader implementation.

#### 2.4.2 Individual variables

Individual variables collected were demographics (age, gender), profession, theoretical preference in treatment approach, amount of training in iCBT, whether they had received advanced training in CBT, years of experience with iCBT and years of experience with psychological therapy in general.

#### 2.4.3 TAM variables

Variables measuring the therapist's acceptance of iCBT were perceived usefulness for the therapist (TPU), perceived usefulness for the patient (PPU), perceived ease of use for the therapist (TPEOU) and perceived ease of use for the patient (PPEOU). In addition, the following antecedent variables were collected: Subjective Norm, Image, Perceptions of external control, Voluntariness and Training (see Table 1 for operationalization).

**Table 1:** Operationalization of TAM variables.

Variable	Operationalization
PU for therapist	The degree to which a user believes that using a particular technology will enhance their job performance or make tasks easier
PU for patients	The extent to which a therapist believes that a particular technology will enhance or improve treatment for the patient
PEOU for therapists	the extent to which a user believes that using a particular technology will be free of effort
PEOU for patients	The extent to which a therapist believes that a particular technology will be free of effort for the patient
Subjective norm	The degree to which an individual perceives that most people who are important to him/her think he/she should use the system
Image	The degree to which an individual perceives that use of an innovation will enhance his or her status in his or her social system
Perceptions of external control	The degree to which an individual believes that organizational and technical resources exist to support the use of the system
Voluntariness	The degree to which an individual believes they can choose whether to use the system as a treatment medium for patients they evaluate
Training	The degree to which a user believes he has received sufficient training to operate the system in line with his or her responsibilities

Note: PU = Perceived Usefulness, PEOU = Perceived ease of use.

The scales used to measure PU and PEOU were translated and adapted from Davis (1989) and Davis et al. (1989). All scales measuring the antecedent variables were translated and adapted from an overview of psychometrically validated items and scales published by Venkatesh and Bala (2008) (see appendix for details regarding translation process and scale adaption).

All scales measuring TAM-constructs were scored on the same 7-point likert-scale used in the original scales, ranging from 1: completely agree to 7: completely disagree (Davis, 1989; Davis et al., 1989; Venkatesh and Bala, 2008).

#### 2.4.4 Contextual variables

The primary contextual variable used in the study was a grouping variable. Group A consisted of four early adopting clinics established in routine care between 2015 and 2018. The establishment of these clinics was initiated and led locally by employees working at each psychiatric hospital in a bottom-up manner, meaning that the decision to adopt iCBT emerged from each outpatient clinic separately without a central mandate or initiative. Furthermore, clinics in group A served as local resources for iCBT and treated patients beyond the catchment area of their psychiatric hospital.

Group B consisted of three clinics established through a dissemination project conducted from 2019 to 2021 within the Western Health Region of Norway. The establishment of the three clinics was conducted as a single implementation project initiated and led centrally through a top-down process, meaning that the decision to adopt iCBT was done by the Western Norway Regional Health Authority, which in turn mandated this adoption in three local psychiatric hospitals. To ensure iCBT coverage throughout the region, the project established one clinic in each of the three hospital trusts which did not have an iCBT resource at the time of dissemination. Furthermore, the therapists working at these clinics received supervision from experienced personnel working as iCBT therapists in one of the clinics in Group A.

Group C consists of 11 clinics established through a similar top-down initiative conducted in the South-Eastern Health Region of Norway from 2020 to 2022. The process was initiated and led centrally by the South-East Regional Health Authority which mandated the adoption and use of iCBT at the 11 clinics. Unlike group B, only the team leaders in charge of the iCBT clinics had received supervision from experienced personnel since project completion. Furthermore, the project did not aim to establish one clinic in each Hospital Trust within the region. Some hospital trusts in the region therefore have no clinics while others have several. The responsibility of the team‑leaders also varies with some being responsible for a single clinic, while others are responsible for multiple clinics located at different areas within the same Hospital Trust.

### 2.5 Ethics

All participants provided informed consent prior to participation. The study was registered in Haukeland University Hospitals project registry (eProtokoll, project ID: 4696–4696) and approved by the Data Protection Officer. As no patient data or sensitive personal information were collected, the study was exempt from review by the Regional Committees for Medical and Health Research Ethics, in accordance with Norwegian regulations on health research (Lov om medisinsk og helsefaglig forskning [helseforskningsloven], 2008, ch. 3; Hovland et al., 2009).

### 2.6 Statistical analysis

Statistical analyses were conducted using SPSS version 26. To address the first and second research question, descriptive statistics (means, standard deviations, frequencies) were calculated for all variables to describe the prevalence and characteristics of the therapists, their workplace contexts, and their acceptance of the iCBT.

To address the third and fourth research question, one-way analyses of variance (ANOVA) were conducted between the groups and the TAM-variables, and factors that distinguished the groups were explored with relation to the group differences found. Since the group sizes varied significantly, and Leven's test indicated significant heterogeneity of variances for several variables (TPU, PPU TPEOU, PPEOU and Voluntariness), we used Welsh's ANOVA with Games-Howell post-hoc tests as they provide more robust estimates with unequal or small group sizes and unequal variances. This approach ensured that the analysis remained statistically valid while maximizing sensitivity to between-group differences.

## 3 Results

### 3.1 iCBT therapist and group characteristics

At the time of data collection there were 18 clinics delivering iCBT in the Norwegian specialist mental health services, employing 67 therapists. Of these, 47 responded to the survey. Two of the participants were excluded because they had not yet started to use iCBT, or because they only worked as a leader and not a therapist. The resulting sample was 45 of the total pool of 67 therapists (67 %), representing all 18 active clinics (see Table 2).

**Table 2:** Descriptive statistics for the overall sample and for each group.

Variable	Total (*N* = 45)	Group A (*N* = 17)	Group B (*N* = 8)	Group C (*N* = 20)
Individual variables				
Age, mean (SD)	41.2 (10.5)	32.8 (6)	44,4 (4,4)	46,7 (10.1)
Gender, n (%)				
Female	31 (68.9 %)	10 (58,8 %)	8 (100 %)	13 (65 %)
Male	13 (28.9 %)	6 (35,3 %)	–	7 (35 %)
Prefer not to say	1 (2.2 %)	1 (5,9 %)	–	–

Profession, n (%)				
Clinical psychologist	18 (40 %)	12 (70,6 %)	–	6 (30 %)
Specialist clinical psychologist	13 (28.9 %)	3 (17,6 %)	4 (50 %)	6 (30 %)
Nurse	1 (2.2 %)	–	–	1 (5 %)
Specialist nurse	6 (13.3 %)	1 (5,9 %)	1 (12,5 %)	4 (20 %)
Social worker	2 (4,4 %)	–	1 (12,5 %)	1 (5 %)
Clinical social worker	2 (4,4 %)	–	2 (25 %)	–
Social educator	2 (4,4 %)	–	–	2 (10 %)
Clinical occupational therapist	1 (2,2 %)	1 (5,9 %)	–	–

Years of experience, mean (SD)				
with iCBT	2.27 (1.68)	2,6 (2,5)	2,7 (0,9)	1,9 (0,8)
with clinical therapy	8.72 (5.67)	5,1 (5,1)	10,3 (4,5)	11,2 (5,1)
Training in iCBT, n (%)				
Both platform and content	35 (77.8 %)	12 (70,6 %)	6 (75 %)	17 (85 %)
Either platform or content	9 (20 %)	5 (29,4 %)	2 (25 %)	2 (10 %)
No training	1 (2.2 %)	–	–	1 (5 %)

Training in CBT, n (%)				
Yes	22 (48.9 %)	5 (29,4 %)	5 (62,5 %)	12 (60 %)
No	23 (51.1 %)	12 (70,6 %)	3 (37,5 %)	8 (40 %)

Preferred therapy approach, n (%)				
None	9 (20 %)	5 (29,4 %)	1 (12,5 %)	3 (15 %)
Cognitive behavioral therapy	20 (44.4 %)	4 (23,5 %)	5 (62,5 %)	11 (55 %)
Metacognitive therapy	8 (17.8 %)	5 (29,4 %)	1 (12,5 %)	2 (10 %)
Acceptance & commitment therapy	1 (22 %)	1 (5,9 %)	–	–
Psychodynamic therapy	2 (4,4 %)	1 (5,9 %)	–	1 (5 %)
Emotion focused therapy	2 (4,4 %)	–	–	2 (10 %)
Dialectiv behavioral therapy	1 (2,2 %)	1 (5,9 %)	–	–
Other	2 (4,4 %)	–	1 (12,5 %)	1 (5 %)

Contextual variables				
Number of clinics, n (%)	18 (100 %)	4 (22.2 %)	3 (16.6 %)	11 (61,1 %)
Accepts self-referral, n (%)	19 (42,2 %)	11 (64,7 %)	8 (100 %)	–
Position size at iCBT clinic, mean % (SD)	32.8 (26.8)	36,8 (34,2)	47,5 (23,2)	23,5 (16,9)
Number of coworkers, mean (SD)	4.38 (1.97)	5,9 (1,7)	4 (1,5)	2,8 (0,8)
Therapy conducted alone, n (%)	22 (48.9 %)	8 (53,3 %)	3 (37,5 %)	13 (68,4 %)

Note: iCBT = Internet-based cognitive behavioral therapy, CBT = Cognitive behavioral therapy, SD = Standard deviation.

Most of the participants were female (68,9 %), educated as clinical psychologists (68,9 %) with a mean age of 41.2 years old (SD = 10,55). On average, participants had 0.33 of their full-time position officially allocated to iCBT (SD = 0.27), although many therapists also reported working with iCBT below and beyond this formal allocation. The average number of coworkers delivering iCBT at each clinic was 4 (SD = 1,97).

Most of the participants (77,8 %) had received training in both the treatment content and the digital platform they used, and about half had also received advanced training in CBT (48,9 %). A third (35.6 %) reported a stronger belief in or preference for a different treatment approach than the one used in the iCBT (i.e. CBT).

Regarding experience as an iCBT therapist, the majority (59 %) had between one and three years of experience (range: 0.25–9 years). Their experience with traditional therapy was more varied with 60 % reporting less than ten years' experience, and the remaining 40 % more than 10 years' experience (range: 0–22 years).

Looking at differences between the groups, group A participants were substantially younger (M = 32.8, SD = 6.0) compared to Groups B (M = 44.4, SD = 4.4) and C (M = 46.7, SD = 10.1). Group B was exclusively female (100 %), while Groups A and C had more balanced gender distributions (58.8 % and 65 % female, respectively).

Professional composition varied considerably across groups. Group A consisted primarily of clinical psychologists (70.6 %), whereas Groups B and C had more diverse professional backgrounds. Despite being younger, Group A participants had comparable iCBT experience (M = 2.6, SD = 2.5) to other groups but significantly less clinical therapy experience (M = 5.1, SD = 5.1) compared to Groups B (M = 10.3, SD = 4.5) and C (M = 11.2, SD = 5.1).

Contextual factors also differed markedly. While all clinics as a standard accepted referrals from a GP, all clinics in Group B also accepted self-referrals (100 %), while 64,7 % of clinics in Group A did the same, and none in Group C. Group A participants worked in larger teams (M = 5.9 coworkers, SD = 1.7) compared to Groups B (M = 4.0, SD = 1.5) and C (M = 2.8, SD = 0.8), and Group C participants were most likely to conduct therapy alone (68.4 %) compared to Groups A (53.3 %) and B (37.5 %).

### 3.2 iCBT therapists acceptance of iCBT and group differences in acceptance

Therapists reported generally high levels of acceptance of iCBT across the TAM constructs (see Table 3). Perceived usefulness and ease of use were rated highly for both therapists and patients (TPU: M = 6.04, SD = 0.78; PPU: M = 5.88, SD = 0.80; TPEOU: M = 5.85, SD = 0.97; PPEOU: M = 5.37, SD = 1.01).

**Table 3:** Acceptance of iCBT across therapist groups: descriptive statistics, reliability, and between-group comparisons.

TAM Variable	Total Mean (SD)	Range	n	α	Group A Mean (SD)	Group B Mean (SD)	Group C Mean (SD)	*p*-value
Perceived Usefulness								
For therapists (TPU)	6,04 (0,78)	4,5–7	6	0.80	5.77 (0.72)	6.77 (0.39)	5.97 (0.78)	< 0.001⁎
For patients (PPU)	5,88 (0,80)	4–7	5	0.74	5.52 (0.64)	6.43 (0.38)	5.98 (0.91)	0.001⁎

Perceived ease of use								
For therapists (TPEOU)	5,85 (0,97)	1,75–7	4	0.79	5.57 (1.18)	6.44 (0.44)	5.85 (0.84)	0.017⁎
For patients (PPEOU)	5,37 (1,01)	3–7	4	0.81	5.12 (0.98)	6.03 (0.43)	5.31 (1.12)	0.005⁎

Antecedent variables								
Subjective Norm	5,93 (0,82)	3–7	3	0.58	6.10 (0.56)	5.92 (0.58)	5.80 (1.06)	0.519
Image	3,04 (1,21)	1–5,67	3	0.86	3.02 (1.41)	3.33 (1.04)	2.95 (1.13)	0.697
Perceptions of external control	6,2 (0,73)	4,5–7	5	0.78	6.35 (0.63)	6.44 (0.65)	5.98 (0.80)	0.210
Voluntariness	5,27 (1,26)	2–7	3	0.47	5.10 (1.34)	4.13 (1.37)	5.88 (0.70)	0.006⁎
Training	6,29 (0,97)	2,5–7	4	0.96	6.22 (0.73)	5.72 (1.43)	6.59 (0.87)	0.210

Note: n = number of items in scale, α = Cronbachs Alpha.

⁎ *p* < .05.

Antecedent variables were also high, including Subjective Norm (M = 5.93, SD = 0.82), Perceived External Control (M = 6.20, SD = 0.73), Voluntariness (M = 5.27, SD = 1.26), and Training (M = 6.29, SD = 0.97).

The only construct with a clearly lower mean score was Image (M = 3.07, SD = 1.21), suggesting they did not associate using iCBT with increased professional status or regard.

Comparing the groups on the TAM-variables, several significant differences were found, including TPU, PPU, TPEOU, PPEOU, and Voluntariness (all *p* < .05; see Table 3).

Post-hoc Games-Howell comparisons revealed that Group B consistently demonstrated the highest scores across measures of usefulness and ease of use, with higher scores than both Group A (M difference = 0.996, *p* < .001) and Group C (M difference = 0.804, *p* = .004) on TPU, and higher scores than Group A on PPU (M difference = 0.907, *p* = .001), TPEOU (M difference = 0.864, *p* = .037) and PPEOU (M difference = 0.914, *p* = .010). However, the largest difference was found in voluntariness where Group C scored significantly higher than Group B (M difference = 1.758, *p* = .019).

No significant group differences were found for Subjective Norm (*p* = .553), Image (*p* = .754), Perceived External Control (*p* = .174), or Training (*p* = .093).

## 4 Discussion

This study provides the first comprehensive national overview of therapists delivering iCBT within Norwegian specialist mental healthcare. Drawing on an extended TAM, we examined therapists' demographic and professional characteristics, their work context, and their acceptance of therapist-guided iCBT. In particular, we explored how these factors varied across clinics established through different implementation approaches, either bottom-up initiatives led locally by clinic staff, or top-down initiatives driven by centralized, external actors.

### 4.1 iCBT therapist characteristics and organizational context

Despite a decade of implementation efforts, only 67 iCBT therapists across 18 clinics work in Norwegian specialist healthcare. This is a striking figure compared to the 10,265 clinical psychologists employed nationally (Statistics Norway, 2024), and shows just how small a part of routine care iCBT is – 10 years after its conception.

While they are few, those that do deliver iCBT in Norway are highly experienced clinicians, with most being clinical psychologists with nearly a decade of clinical experience. At the same time, they represent a heterogeneous group with different professional backgrounds, theoretical preferences, and work environments, indicating that the adoption, acceptance and use of iCBT is not limited to a minority of therapists, but most – even those with considerable experience as face-to-face therapists.

The iCBT services are largely organized in a decentralized manner, with some clinics serving as supervisory units. This organizational model appears uncommon compared to centralized clinics delivering treatments to larger catchment areas or even nationally (Andersson and Hedman, 2013; Hadjistavropoulos et al., 2016). While decentralized delivery can yield similar treatment effects (Hadjistavropoulos et al., 2016), Gervind et al. (2024) found that therapists at decentralized clinics showed significantly lower adherence to iCBT treatments than those in centralized clinics, and centralized units demonstrated better implementation sustainability. For the time being, differences in adherence between the clinics in Norway remains unknown. Regarding sustainability though, the decentralized organization seem to work, given the continued operation and addition of new clinics over the preceding decade.

### 4.2 Acceptance of iCBT and the role of image

Across the sample, therapists reported high levels of acceptance of iCBT. Both perceived usefulness and ease of use were rated highly for patients and therapists, reinforcing previous findings that iCBT can be seen as both clinically effective and operationally feasible (Hadjistavropoulos et al., 2020; Hedman-Lagerlöf et al., 2023). Subjective norm, Voluntariness, Perceptions of external control, and Training were also rated highly, suggesting that iCBT has become well integrated into the organizational culture and support structures of specialist care. This high acceptance is present in all groups, despite them differing in implementation approach, organization and employees.

One reason why the acceptance in our sample is high may be the training the participants have received in iCBT. Almost 80 % report thorough training in iCBT and that their training was sufficient to use the iCBT. In line with this, Terpstra et al. (2018) found that therapists with no former iCBT training scored perceived usefulness and ease of use highly after a structured training session.

Previous studies looking at therapists who do not use iCBT tend to show that therapists are skeptical to it replacing face-to-face therapy and report low likelihood of use (Hildebrand et al., 2024; Musiat et al., 2014). However, some studies also show that factors such as age or therapeutic orientation could influence their attitudes with older therapists perceiving online therapy as less effective (Rutkowska et al., 2023) and therapists with an orientation towards cognitive behavioral therapy or towards systemic therapies as being more accepting of using technology with therapy. Considering the variation in our sample, our data does not seem to show the same trends. Instead, high acceptability is present across several therapist characteristics indicating that iCBT could be acceptable for most working with therapy, if they have used it. This possibility of prior use as a positive influence on therapists' attitudes has also been reported in previous studies (Békés et al., 2021; Farrer et al., 2023). Furthermore, although the TAM posits that forced adoption of an innovation can lead to reduced motivation (Venkatesh and Bala, 2008), it has also been found that mass exposure and hands on experience, even when forced, often can lead to more positive perceptions over time (Békés et al., 2021). If this is the case, user characteristics and organization may not be a primary concern in implementation strategy development. Rather, a major barrier may lie in the low exposure to actual use among therapists.

While the participants' scores on most TAM-variables were high, Image was scored markedly lower, suggesting that the therapists use the iCBT with the explicit expectation to lose professional regard or social status. This finding echoes research suggesting digital therapies may lack cultural prestige within traditional mental health services where in-person work remains the gold standard (Andersson et al., 2019; Chong et al., 2022).

The reason for low scores on Image in our sample remains unclear. Image may be higher among therapists using telephone-supported iCBTs, or among therapists working in a different country. However, if this finding persists across contexts, addressing and improving Image is likely to improve adoption and implementation.

Low image may stem from therapists perceiving iCBT as requiring less skill and effort than delivering face-to-face treatments. The idea that effort or difficulty somehow affect our understanding of somethings value or quality is well documented in several cognitive phenomenon and biases. For example, the Effort Heuristic demonstrates that people judge quality based on perceived effort invested (Kruger et al., 2004). Similarly, the IKEA Effect shows that people value products requiring personal effort, like self-assembled furniture (Norton et al., 2012).

A second reason may be that iCBT conflict with important professional identities (Cresswell and Sheikh, 2013; Lee et al., 2006). For example, some therapists may view therapeutic standardization as incompatible with their understanding of therapy or clinical practice. However, many aspects of iCBT align well with traditional clinical roles. Highlighting these compatible elements when training clinicians could frame iCBT as congruent with valued professional identities, boosting image (Lee et al., 2006). An example of this could be to showcase the skill requirements that are unique for iCBT therapists, like the ability to deliver therapeutic support without undermining the patient's responsibility to complete important therapeutic steps on their own, or the ability to use clear and concise language without the support of nonverbal cues. Although focusing on the importance of therapists, their skill and the effort they expend to deliver an iCBT treatment may not be the framing that promotes adoption among the health care administration, it could improve Image among clinicians.

Importantly, while loss of Image does not seem to hinder acceptance among experienced users of iCBT, it may be a major barrier to implementation among therapists with no actual user experience. Given that professional identity and clinical values are known to influence the uptake of new practices (Cresswell and Sheikh, 2013), this underlines the need to address cultural as well as technical barriers in digital health implementation.

### 4.3 Group differences in acceptance

While the acceptance levels where high overall, important group differences also emerged. Group B therapists, working in clinics established through structured top-down dissemination with ongoing supervision, consistently reported the highest scores across perceived usefulness and ease of use. This aligns with literature showing that centralized implementation support, supervision, and resource allocation improve therapist buy-in for digital interventions (Molloy et al., 2021; Tremain et al., 2020; Vis et al., 2023).

Group B therapists also scored lowest on Voluntariness showing that a greater sense of autonomy did not correspond with higher acceptance. This supports findings from broader technology adoption literature suggesting that voluntariness alone does not drive sustained use if not accompanied by adequate training and support (Davis et al., 2024; Garavand et al., 2016; Venkatesh and Bala, 2008). Greater autonomy may protect against resistance but does not in itself foster engagement.

## 5 Limitations

Several limitations should be considered when interpreting these findings. First, the sample size of 45 therapists provided limited statistical power to detect small-to-moderate differences between the groups, potentially leading to Type II errors for meaningful associations. This constraint also precluded more sophisticated analyses such as structural equation modeling or multiple regression that could have provided greater insight into the relative importance of different predictors and potential mediating pathways. While Levene's ANOVA was employed to address assumptions with small sample sizes, the risk of overlooking clinically relevant differences remains. However, the sample represents 67 % of all iCBT therapists in Norway, providing strong external validity and ensuring that findings reflect the experiences of the majority of iCBT-therapists in Norway.

Second, the cross-sectional design prevents causal conclusions about the relationships between therapist characteristics, contextual factors, and iCBT acceptance. Although longitudinal data would strengthen causal inferences, cross-sectional associations remain valuable for identifying factors associated with technology acceptance and can inform the development of targeted interventions to support implementation.

Third, reliance on self-reported data introduces potential sources of bias, including social desirability bias in the responses and systematic response patterns that may either inflate positive attitudes or minimize implementation challenges. While such biases are inherent limitations of survey methodology, self-report remains the most practical approach for capturing subjective experiences and attitudes at scale, particularly for constructs like acceptance and perceived barriers that are inherently subjective.

Finally, unmeasured factors such as leadership support, workload pressures or organizational culture may explain some of the variation observed between groups. For instance, while the data show whether the therapists received advanced training in a therapeutic approach like CBT, it lacks information about the nature and quality of this training, limiting the conclusions we can draw. Nevertheless, examining how iCBT was established and implemented within each group offers valuable insights into factors that influence iCBT adoption. Future research using mixed-methods approaches could illuminate additional determinants and provide a more complete understanding of these dynamics.

## 6 Theoretical and practical implications

This study demonstrates the value of the Technology Acceptance Model (TAM) as a conceptual framework for understanding therapist acceptance of digital mental health interventions in real-world clinical settings. A key finding was that therapists expected to lose professional regard from peers by using iCBT. This suggests that professional regard may be a significant barrier to widespread use. Furthermore, experiential, rather than informational approaches, could be useful to progress normalization. Shaping the professional identity as a therapist to be in line with iCBT use could be done by embedding digital mental health competencies as a standard part in all therapist training curricula, expanding access to or mandating use of iCBT to allow therapists direct experience with its clinical utility, and implementing behavioral-first change strategies that follow behavioral theories suggesting practice changes can shift attitudes over time among initially skeptical practitioners.

Future research should investigate differences in acceptance between active iCBT users versus those merely informed about it, examine the Image construct as a professional culture barrier requiring targeted intervention and develop patient-centered acceptance measures since therapists may prioritize perceived patient benefits over personal utility.

TAM's utility in technology adoption is highly supported, but healthcare applications require attention to mechanisms specific to healthcare professionals, their roles, and the dual-user nature of therapeutic technologies. Therapists' roles may encompass norms regarding how therapy should be done (i.e. face-to-face), but also norms regarding their role as a health care professional prompting them to prioritize the treatment approaches they believe is most helpful for their patients. Understanding which aspects of these professional roles to target through implementation strategies may be critical for successful digital mental health adoption. Future research should therefor aim at refining technology acceptance models to better capture these idiosyncrasies.

## 7 Conclusion

Nearly ten years after its introduction, iCBT remains underused in Norwegian specialist mental healthcare, with only 67 therapists across 18 clinics delivering it. Using the Technology Acceptance Model, this study found high therapist acceptance overall. Clinics where the use of iCBT was initiated through structured top-down processes lead by external personnel with weekly supervision by experienced therapists reported highest acceptance, indicating that high acceptance is possible even with mandated implementation. However, iCBT was not seen as enhancing professional status, suggesting a cultural barrier that may limit wider adoption. Improving the image of digital therapy and ensuring long-term support may be key to scaling iCBT more effectively.

## Abbreviations

iCBT internet-delivered cognitive behavioral therapy.

TAM The Technology Acceptance Model.

BI Behavioral intention.

PU Perceived usefulness.

PEOU Perceived ease of use.

TPU Perceived usefulness for the therapist.

PPU Perceived usefulness for the patient.

TPEOU Perceived ease of use for the therapist.

PPEOU Perceived ease of use for the patient.

## CRediT authorship contribution statement

The study was conceptualized and designed by RN, RK and CV. Data collection was carried out by RN and RK. RN conducted the data analysis and all authors interpretation. RN drafted the manuscript. CV and RK critically reviewed and revised the manuscript for important intellectual content. All authors read and approved the final version of the manuscript.

## Disclaimer

The authors alone are responsible for the content in this article. These do not necessarily reflect the policies or positions of the funding bodies involved.

## Role of sponsor

This study was funded by The 10.13039/501100005416Norwegian Research Council (Norges forskningsråd), P.O. Box 564 NO-1327 Lysaker, Norway. The funding body played no role in the design of the study, the collection, analysis, and interpretation of data or in writing the manuscript.

## Declaration of generative AI and AI-assisted technologies in the writing process

During the preparation of this work the author(s) used ClaudeAI (large language model developed by Anthropic) in order to improve readability, to identify and remove redundant or unnecessarily lengthy text, and to create tables. After using Claude, the author(s) reviewed and edited the content as needed and take(s) full responsibility for the content of the published article.

## Funding

The 10.13039/501100005416Research Council of Norway (Norges forskningsråd), Grant number: 309264.

## Declaration of competing interest

The authors declare that they have no known competing financial interests or personal relationships that could have appeared to influence the work reported in this paper.

## Appendix

### Adaptation and translation of TAM-Scales

In line with previous research (Venkatesh, 2000; Venkatesh and Davis, 2000), all scales measuring TAM-constructs were adapted to relate specifically to the technology in question. This entailed that PU and PEOU were expanded and split into two constructs each, one regarding the therapists' perceptions of how the technology would work for them as therapists, and one regarding the therapists' perceptions of how the technology would work for their patients. After the adaptation and translation to Norwegian, an expert panel was used to evaluate how well the adapted items fit the original operationalisation of the constructs. The expert panel consisted of four persons. Two of the experts where clinical psychologists with experience from ICBT treatment and had a PhD in clinical psychology. One expert was a psychiatric nurse with experience from ICBT treatment and as a leader of an ICBT clinic. The final expert was a research consultant with previous experience from research on ICBT and implementation. All experts were informed about the aim of the panel and signed informed consents. The panel discussions were done face-to-face and recorded with both video and audio. All experts were informed of the purpose of the expert panel and how the recordings would be used before signing informed consent forms.

All experts were first given a questionnaire with the adapted items for each construct and asked to answer them as if they were therapists. They were asked to note if any of the items were difficult to understand, poorly worded or if they were ambiguous. Feedback on the items were then given by each in turn and discussed. After this initial feedback, the experts completed a card-sorting task where they matched groups of items to the constructs and their definition. All grouped items were correctly matched with their construct by all the experts. Finally, the panel discussed whether any items should be added, removed, or rephrased to better fit the operationalisation of the constructs.

All scales were adapted according to the feedback from the expert panel, and the experts again gave independent feedback on the scales before they were mounted as a digital questionnaire. Finally, the digital questionnaire was tested, and the user interface was adjusted to assure that the digital scales worked as intended.
